# Senolytic Therapy: A Potential Approach for the Elimination of Oncogene-Induced Senescent HPV-Positive Cells

**DOI:** 10.3390/ijms232415512

**Published:** 2022-12-08

**Authors:** Tareq Saleh, Ashraf I. Khasawneh, Nisreen Himsawi, Jumana Abu-Raideh, Vera Ejeilat, Ahmed M. Elshazly, David A. Gewirtz

**Affiliations:** 1 Department of Pharmacology and Public Health, Faculty of Medicine, The Hashemite University, Zarqa 13133, Jordan; 2 Department of Microbiology, Pathology, and Forensic Medicine, Faculty of Medicine, The Hashemite University, Zarqa 13133, Jordan; 3 Department of Anatomy and Histology, Faculty of Medicine, The University of Jordan, Amman 11942, Jordan; 4 Department of Pharmacology and Toxicology, School of Medicine, Virginia Commonwealth University, Richmond, VA 23298, USA; 5 Department of Pharmacology and Toxicology, Faculty of Pharmacy, Kafrelsheikh University, Kafrelsheikh 33516, Egypt; 6 Massey Cancer Center, Virginia Commonwealth University, Richmond, VA 23298, USA

**Keywords:** senescence, oncogene-induced senescence, HPV, senolytics, cervical cancer

## Abstract

Senescence represents a unique cellular stress response characterized by a stable growth arrest, macromolecular alterations, and wide spectrum changes in gene expression. Classically, senescence is the end-product of progressive telomeric attrition resulting from the repetitive division of somatic cells. In addition, senescent cells accumulate in premalignant lesions, in part, as a product of oncogene hyperactivation, reflecting one element of the tumor suppressive function of senescence. Oncogenic processes that induce senescence include overexpression/hyperactivation of H-Ras, B-Raf, and cyclin E as well as inactivation of PTEN. Oncogenic viruses, such as Human Papilloma Virus (HPV), have also been shown to induce senescence. High-risk strains of HPV drive the immortalization, and hence transformation, of cervical epithelial cells via several mechanisms, but primarily via deregulation of the cell cycle, and possibly, by facilitating escape from senescence. Despite the wide and successful utilization of HPV vaccines in reducing the incidence of cervical cancer, this measure is not effective in preventing cancer development in individuals already positive for HPV. Accordingly, in this commentary, we focus on the potential contribution of oncogene and HPV-induced senescence (OIS) in cervical cancer. We further consider the potential utility of senolytic agents for the elimination of HPV-harboring senescent cells as a strategy for reducing HPV-driven transformation and the risk of cervical cancer development.

## 1. Introduction

Cellular replicative senescence was first described by Leonard Hayflick and Paul Moorhead more than five decades ago as a stable exit from the cell cycle in non-transformed fibroblasts [1,2]. Senescence is a specialized form of growth arrest that plays a dynamic role in mediating multiple physiological and pathological processes [3]. The senescent growth arrest is stable and durable, in that, senescent cells are unresponsive to mitogenic drivers, but remain viable and metabolically active [4]. Replicative senescence represents the classical response to telomeric dysfunction that occurs due to the “end replication crisis” in dividing eukaryotic cells [5]. In addition to preventing the proliferation of cells containing dysfunctional telomeres, senescence is induced in response to other stimuli that also have the potential to promote malignant transformation, including oxidative and genotoxic stress, the latter often precipitated by exposure to DNA- damaging drugs or ionizing radiation [6]. With reference to the focus of this article, senescence is also a well-established response to oncogene overexpression [7], thereby presenting a fundamental barrier to malignant transformation [8].

In support of senescence as a tumor suppressive mechanism, senescent cells have been shown to accumulate in premalignant lesions [9]. The classical example is the identification of senescence markers in naevi containing B-Raf overexpressing melanocytes [10]. In addition to B-Raf, the activation of several other oncogenes, such as Ras, Akt, and E2F, or conversely, the inactivation of certain tumor suppressor genes, such as PTEN, can also promote senescence in somatic cells [11]. In most cases, senescence is sufficient to block oncogenic transformation by conferring a permanent abrogation of growth in cells harboring an active oncogene; consequently, bypassing oncogene-induced senescence is essential for malignant transformation. Nevertheless, the co-expression of an additional oncogene or inactivation of a tumor suppressor gene, or a “second hit”, is generally accepted as being required for a cell to overcome (or escape) Oncogene-Induced Senescence (OIS) and proceed towards malignancy. However, the function of senescence in transformation is likely to be more complex, as recent evidence has suggested that the accumulation of oncogene-induced senescent cells contributes to disease progression through cell-non-autonomous effects [12]. While OIS is not directly related to telomeric dysfunction, it is also a product of DNA damage which results when cells are undergoing abnormally rapid replication due to oncogene-driven proliferative stimulation [13]. Despite the spectrum of senescence-inducing insults, the senescent phenotype shares several prominent features, as detailed below. Nonetheless, it is essential to note that senescent cells are highly heterogeneous, with extensive diversity in their gene expression profiles and the manifestation of senescence markers [14].

Human papillomavirus (HPV) infection is the leading cause of cervical cancer [15]. It has been established that infection with high-risk strains of HPV, namely, HPV-16 and HPV-18, is associated with senescence induction, a process largely dictated through the actions of the viral oncoproteins E6 and E7 (both suppression of E6 or E7 by E2 in HPV-infected cells or transfection of naïve cells with E7) [16,17,18]. Both E6 and E7 are considered essential factors for carcinogenesis in HPV-positive cells, as the continuous expression of these oncoproteins plays a key role in the induction and maintenance of the cellular malignant features [16]. E6 and E7 expression is associated with the degradation of the tumor suppressors p53 and retinoblastoma (Rb) proteins, respectively [19]. Conversely, E6 suppression induces p53 expression and upregulates the cyclin-dependent kinase inhibitor p21^Cip1^, leading to cell cycle arrest and senescence induction [20]. Similarly, the suppression of E7 induces Rb expression and the formation of the Rb-E2F complex leading to transcriptional repression and cell cycle arrest [20]. Thus, HPV infection represents a potential pathway to OIS, making the accumulation of HPV-positive senescent cells in cervical lesions a likely precancerous process. Consequently, targeting either E6 and/or E7 oncoproteins in cancerous cells or senescent cells, represents a potential strategy for the suppression of HPV-positive tumor cell growth, thereby reducing the risk of cervical cancer in infected individuals [21]. In this commentary, we provide an overview of OIS, and its complex contribution to cancer development, and, more specifically, discuss HPV-Induced Senescence as a variant of OIS. Moreover, we propose the use of senolytics, a newly emerging drug class that selectively targets senescent cells, as a novel approach for the mitigation of the progression of premalignant lesions toward cancer, including HPV-infected Cervical Intraepithelial Neoplasia (CIN).

## 2. Oncogene-Induced Senescence

### 2.1. Hallmarks of Senescence

Aside from the durable growth arrest, which is considered the primary characteristic of senescence, senescent cells exhibit a plethora of features that collectively represent the senescent phenotype [22]. The senescence characteristics are both cell-autonomous and cell-non-autonomous [23]. Intrinsically, senescent cells exhibit an enlarged and flattened morphology, chromatin rearrangement known as senescence-associated heterochromatic foci (SAHFs) [24], which are frequently associated with OIS, and enhanced expression of the senescence-associated β-galactosidase (SA-β-gal) activity [25], which reflects increased lysosomal content [26]. Furthermore, senescent cells are characterized by the accumulation of reactive oxygen species (ROS) coupled with ROS-mediated macromolecular damage [27]. In addition, under persistent activation of the DNA damage repair response (DDR), senescent cells display nuclear foci termed DNA segments with chromatin alterations reinforcing senescence (DNA-SCARS); these are crucial elements that further sustain the senescent state due to the activation of DDR proteins such as p53 [18,28]. Another essential feature defining senescent cells is the production of a diverse range of cytokines, chemokines, extracellular matrix proteases, growth factors, and other signaling molecules, collectively termed the senescence-associated secretory phenotype (SASP), which largely mediates the extrinsic effects of senescence [29,30]. However, none of these features is uniquely representative or specific to senescence as they can also be observed in other forms of cellular stress or cell cycle arrest [22]. Accordingly, senescent cells are usually identified by the examination of a profile of multiple senescence-associated biomarkers [31] (Table 1).

#### 2.1.1. Growth Arrest

Senescence activation is commonly observed at the G_1_ phase of the cell cycle, but cell growth may also be arrested in the G_1_, S, or G_2_/M phases of the cell cycle [47]. The senescent growth arrest is governed by several cell cycle regulators, primarily the p53/p21^Cip1^ and p16^INK4a^/pRb pathways, which are activated upon entry into senescence [48,49]. The essential role of p53 in regulating the senescent growth arrest stems from the transcriptional upregulation of the Cyclin-dependent Kinase Inhibitor (CDKI) p21^Cip1^. In order to prevent the proliferation of damaged cells, the tumor suppressor protein p53 transactivates its target genes, whose gene products promote either apoptosis to remove damaged cells from the body and/or cell cycle arrest and DNA damage repair mechanisms to mitigate genotoxic injury [50,51,52]. p53 binding can repress the expression of several genes including survivin, CDC25C, CDC25B, CHK2, cyclin B, CKS1B, RECQL4, and cdc20 while enhancing the transcription of p21^Cip1^ [51,53]. p21^Cip1^ inhibits the activity of various cyclin-dependent kinases (CDKs) by binding and blocking the ATP binding site of CDKs, subsequently preventing CDK phosphorylation and interfering with cell cycle progression [51,54,55]. p21^Cip1^ inhibition of cyclin D-CDK4/6 complexs activation prevents Rb phosphorylation and enhances Rb-E2F complex sequestration. In proliferating cells, Rb is hyperphosphorylated by CDK/cyclin complexes, allowing the release of the transcription factor, E2F, from the Rb-E2F complex, and transcription of key S-phase genes [56]; hence binding of Rb to E2F represses the transcription of cell cycle genes such as hTERT, EZH2, and CHKI [50,57]. Despite the critical role of p21^Cip1^ in mediating the senescent growth arrest, the deletion of p21^Cip1^ is not sufficient to prevent mouse fibroblasts from undergoing a proliferative arrest upon senescence induction, indicating that p21^Cip1^ is not absolutely required for the senescent growth arrest to occur [58]. This is true, in part, because growth arrest can be mediated through the p16^INK4a^/Rb pathway [58,59]. p16^INK4a^, an inhibitor of CDK4 and CDK6, is more closely associated with senescence induction and is considered a significant activator of Rb [33,48,60].

Similarly to p21^Cip1^, p16^INK4a^ is a CDKI that interferes with cyclin D-CDK4/6 complexes, maintaining Rb-E2F binding, and stabilizing a G_1_/S cell cycle arrest by activating the pRb checkpoint [48,54,60]. p16^INK4a^ is classically activated in senescent cells and is frequently utilized as a characteristic marker to identify senescent cells in a variety of senescence models [61]. Moreover, it has been demonstrated that cells that are positive for p16^INK4a^ often display other markers of senescence including SA-β-gal, altered morphology, and increased expression of SASP (discussed below) [61]. Accordingly, and since the senescent growth arrest, particularly that associated with OIS, interferes with the progression of cells at risk of malignant transformation, p16^INK4a^ is considered to function as a tumor suppressor gene [62,63]. Moreover, the *CDK2A* locus encoding for p16^INK4a^ is frequently inactivated through deletions, point mutations, or hypermethylation in many malignancies including melanoma, leukemia, pancreatic and head and neck carcinomas, reflecting its established tumor suppressor function [64]. Importantly, as p16^INK4a^ is expressed in several types of senescent cells, its overexpression is also considered a major hallmark of HPV-associated cancers, including HPV-positive cervical carcinomas and HPV-positive head and neck cancers (see section below) [20,65]. In HPV-positive cancers, the virus-encoded oncoprotein, E7, mediates the inactivation of Rb, resulting in the release of E2F from the inhibitory complex and entry into the S phase [66]. In order to compensate for the loss of Rb function, cells increase expression of p16^INK4a^ in effort to suppress cell cycle progression [66].

Senescent growth arrest is unique in that it is highly stable and durable. Unlike quiescent cells that can resume proliferation when favorable growth conditions are restored, senescent cells are unresponsive to growth stimuli [67]. It should also be emphasized that quiescent cells do not demonstrate the hallmarks of senescence. Furthermore, senescent cells preserve their active metabolic status, albeit dysregulated, as evidenced by a remarkable increase in oxygen consumption rate and mitochondrial oxidative metabolism in OIS cells as opposed to quiescent cells which typically have reduced metabolic rates [41]. Nevertheless, recent evidence has demonstrated that certain forms of senescence can be overcome, and that senescent cells, especially tumor cells induced into senescence by exposure to cancer therapy, can resume proliferation [68]. For example, H1299 non-small cell lung cancer cells exposed to camptothecin can regain proliferative ability upon discontinuation of drug exposure, although recovery from the senescent growth arrest was a rare event (approximately, 1 in 10^6^ cells) [69]. Our own laboratory has also previously demonstrated that several tumor cell types (specifically, H460 lung, HCT116 colon, HN30 head and neck, Myc-CaP prostate, and 4T1 breast cancer cells) induced into senescence by exposure to chemotherapy or ionizing radiation can overcome the senescent growth arrest following short-term drug or radiation treatment [70,71,72].

It is noteworthy that conditional p53 inactivation or interference with SAHF formation can be permissive for the escape from doxorubicin-induced senescent growth arrest in tumor cells [73]. While the likelihood of the escape from the stable senescence-induced growth arrest is largely represented in tumor cell models, the reversal has also been observed in BJ cells induced into senescence by replicative exhaustion [74]. The stability of the senescent growth arrest is partly dependent on the expression of p16^INK4a^ or p53, as their loss of function can be associated with a less stable form of growth arrest. For instance, senescent BJ cells, which have relatively low expression of p16^INK4a^, can re-enter the cell cycle upon p53 inactivation, in contrast to the more stable senescence in WI38 fibroblasts, where the p16^INK4a^/Rb pathway tends to be fully active [33,74]. The reversibility of senescence-mediated growth arrest has been also documented in OIS models including Ras-induced senescent pancreatic cells, Akt1-induced senescent pancreatic cells, and oncogene-induced senescent astrocytes [75,76,77]. The potential for escape from OIS is consistent with the premise that oncogene-overexpressing cells are likely to utilize this strategy to progress into a state of malignancy.

#### 2.1.2. Morphological and Macromolecular Changes

Senescent cells are characterized by distinct morphological features. Specifically, senescent cells exhibit an enlarged, flattened appearance and irregularly-shaped nuclei [36]. The increase in cellular size is frequently accompanied by an increase in the size of the nucleus and nucleoli [78]. Senescent cells also develop numerous cytoplasmic vacuoles, increased numbers of cytoplasmic microfilaments, enlarged lysosomal bodies, and prominent Golgi apparatuses [42,79]. Moreover, senescent cells have enhanced lysosomal biogenesis, which is marked by increased expression of SA-β-gal, the most frequently used marker for the identification of senescent cells [80]. The accumulation of lipofuscin aggregates, proteins that accumulate progressively in the lysosomes of aged, post-mitotic cells, was also established as a hallmark of senescent cells [40,81,82,83].

#### 2.1.3. DNA Damage

Persistent DNA damage is the most consistent trigger of senescence. Classically, as cells become replicatively exhausted, their telomeres reach a critical length and fail to bind telomere-capping proteins. In brief, a strip of telomeric nucleotides is excised each time a proliferating cell completes a cycle of DNA synthesis in preparation for mitosis [5]. Upon repetitive loss of the 6-base sequence of telomeric DNA, the cell reaches a point where further division cycles will result in chromosomal injury [84]. In fact, telomere attrition can lead to a number of DNA lesions including end-end chromosomal fusions, breakage of anaphase chromatin bridges, and translocations. However, it is the altered status of shortened telomeres, rather than the mere loss of telomeric DNA, that is directly linked to replicative senescence [37]. These dysfunctional telomeric ends are then recognized as “exposed” ends of DNA [85], subsequently activating the DDR pathways and leading to the prompt activation of cell cycle blockers such as p21^Cip1^ [86]. Importantly, dysfunctional telomeres continue to possess enough content of telomere-binding proteins to inhibit DNA repair, and thus, maintain a persistent DNA damage drive [87,88]. Similarly, oncogene activation results in hyperproliferation as well as a high degree of replicative stress, subsequently leading to the accumulation of single and double-stranded DNA breaks [89,90]. Double-stranded DNA breaks (DSBs) are a major activator of DDR, initiating autophosphorylation and activation of ATM, which then drives histone H2AX phosphorylation, facilitating DNA repair complex formation [6]. Moreover, ATM activation results in downstream phosphorylation of p53 and subsequent induction of p21^Cip1^, which mediates the senescent growth arrest [91].

DNA damage responses play an essential role in the activation of oncogene-induced senescence, given that inactivation of key proteins involved in DDR results in evasion of the senescent phenotype, continued proliferation, and malignant transformation [13,92]. Persistent DNA damage in senescent cells can be irreparable and associated with lesions encompassing PML nuclear bodies, lack of activation of the established DNA repair proteins, such as RAD51, but with activated p53 and CHK2, and suppressed DNA synthesis. These lesions are more likely to describe senescent cells in a stable growth arrest, and are collectively termed DNA segments with chromatin alterations reinforcing senescence (DNA-SCARS) [18]. Interestingly, and relevant to this review, artificial expression of the HPV oncoprotein, E7 in HCA2 cells results in the formation of DNA-SCARS, consistent with the ability of HPV to induce senescence [18]. Collectively, DNA damage and the activation of the DDR are major hallmarks of senescence, including OIS [13].

#### 2.1.4. Mitochondrial Dysfunction

Senescent cells often accumulate damaged mitochondria characterized by (i) reduced oxidative potential, (ii) decreased mitochondrial membrane potential, (iii) structural changes, and (iv) build-up in free radicals [93,94]. In addition to being a component of replicative and therapy-induced senescence, mitochondrial dysfunction is frequently observed in models of OIS [95]. For example, upon Ras overexpression in normal human fibroblasts, mitochondria increase in mass, potentially due to reduced mitophagic turnover [96], and accumulate ROS leading to oxidative DNA injury [95]. Moreover, the induction of Ras reduces the energy-generating capacity of mitochondria marked by low ATP production [95]. However, unlike what occurs in aging-associated senescent cells, NAD+, which plays an important role in mitochondrial redox reactions, is increased rather than decreased (thus shifting the NAD+/NADPH balance up) in OIS models [97]. Still, in oncogene-induced senescent cells, the accumulation of ROS due to mitochondrial dysfunction appears to be necessary for gene expression changes leading to the activation of the SASP, which are largely mediated by the nuclear factor kappa B (NFκB) pathway [27]. Lastly, the role of mitochondria in regulating Ca^+2^ homeostasis is perturbed in senescent cells, and in fact, might facilitate the escape from OIS [98]. Interestingly, components of oncogenic HPV proteins can localize in juxtaposition to the mitochondrial inner membrane causing morphological changes and facilitating increased production of ROS [99]. This provides a possible link between HPV infection, the development of mitochondrial dysfunction, and OIS.

#### 2.1.5. Epigenetic Changes

In addition to their constitutive structural roles, in senescent cells, heterochromatin and euchromatin exhibit distinct post-translational modifications of their histone proteins and associate with different sets of facultative chromatin binding proteins, together termed as Senescence-associated heterochromatin foci (SAHF) [43]. SAHF were first described by Scott Lowe and coworkers after observing that DAPI-stained senescent human cells displayed a relatively diffuse distribution of DNA throughout the nucleus, which appeared as bright, punctate DNA foci, and that the chromatin in these foci appeared more compact than during the normal interphase of growing cells [43]. Each SAHF might consist of a single condensed chromosome that is enriched with histone modifications and proteins that are associated with epigenetically silenced genes such as the E2F family [100]. SAHF mediate epigenetic regulation during OIS; however, SAHF are not considered a common feature of cellular senescence as they are not universally observed in cells undergoing replicative senescence and are rarely observed in vivo [100].

Histone edits involved in SAHF include trimethylation of histone H3 at lysine 9 (H3K9Me3) and its binding partner heterochromatin protein 1 (HP1) [42], and the persistent phosphorylation of the DNA DSBs marker H2AX (γH2AX) [42,100]. Functionally, SAHF repress the expression of proliferation-linked genes, such as cyclin A, thereby contributing to senescence-associated cell cycle arrest. Evidently, interference with the ability of senescent cells to condense their chromatin and to generate some of these classical histone modifications, such as H3K9Me3, can facilitate the escape from the senescent growth arrest [73]. Lastly, a functional pRB/p16^INK4a^ pathway is required for the efficient formation of SAHF [101,102].

#### 2.1.6. Resistance to Apoptosis

Senescent cells tend to be resistant to apoptosis. A potential mechanism for this characteristic of senescent cells is the upregulation of the anti-apoptotic members of the BCL-2 family [103]. More specifically, senescent cells (whether by replicative exhaustion, chemotherapy, radiation, or oncogene overexpression) appear to be largely dependent on BCL-X_L_ for their survival, since the genetic or pharmacological inhibition of BCL-X_L_ (and, to a lesser extent, BCL-W) results in selective and immediate induction of apoptosis in these cells [104]. However, in other studies comparing young cells and senescent cells exposed to H_2_O_2_, despite the low levels of BCL-2 in the senescent cells, they were still more resistant to apoptosis compared to their young counterparts [105]. Thus, the decrease of BCL-2 during cellular aging has no apparent impact on induced death in senescent cells. Other studies suggest that senescent cells being in a non-cycling state, along with increasing levels of CDK inhibition, may alternatively explain their resistance to cell death [105]. MCL-1 is another member of the BCL-2 family that plays a role in maintaining the survival of senescent cells since its selective inhibition commits these cells to cellular demise [106]. Another potential explanation of how senescent cells resist cell death is by downregulating the expression of caspase-3, which is responsible for the execution of apoptosis upon the mitochondrial release of cytochrome c and activation of the caspase pathway [107].

Recent evidence has also suggested a role for the Forkhead box O (FOXO4)-p53 interaction in the maintenance of senescent cell survival [108]. FOXO4 is part of a family of transcription factors that are involved in regulating gene expression of several cell survival pathways. Moreover, FOXO4 has been described to regulate senescence-associated pathways, especially in models of OIS. For example, BRAF^V600E^ expression, which routinely triggers OIS, results in ROS build-up, JNK activation, and eventually, phosphorylation and activation of FOXO4 [109]. FOXO4, in turn, is capable of inducing a stable senescent growth arrest via upregulation of p21^Cip1^ (another p53 downstream target) expression [109]. The interaction between FOXO4 and p53 at sites of DNA damage appears to be responsible for shifting cell fate from apoptosis to senescence. Baar et al. showed that radiated senescent fibroblasts upregulate FOXO4. Once FOXO4 is inhibited prior to radiation exposure, cells instead undergo classical cytochrome c-mediated mitochondrial apoptosis, thus reflecting an important role for FOXO4 in rendering senescent cells resistant to cell death [110]. The importance of FOXO4-p53 interaction in preventing apoptosis in senescent cells has been confirmed in studies by Le et al., which showed that the selective targeting of FOXO4-p53 robustly kills senescent cells [111]. Overall, these mechanisms appear to account for the ability of senescent cells to persist for prolonged periods in vivo, including in premalignant lesions upon oncogene overexpression.

#### 2.1.7. The SASP

In contrast to quiescent cells, senescent cells modulate their microenvironment via the secretion of proinflammatory chemokines, cytokines, growth factors, and proteases, collectively termed the senescence-associated secretory phenotype or SASP [112]. Indeed, the SASP represents a double-edged sword. On one hand, the SASP directly contributes to an insidious inflammatory process that triggers immunosurveillance of senescent cells [29], while on the other hand, the SASP can facilitate the ability of a subpopulation of senescent cells to evade immunorecognition [113,114]. Transcription of the SASP inflammatory genes is regulated primarily by two well-established transcription factors NF-κB and CCAAT-enhancer binding protein β (C/EBPβ) [115]. Analysis of the senescent cell chromatin identified the NF-kB subunit p65 (also known in humans as the *RELA* gene) as a major component of the transcription machinery that leads to the expression of cytokines and chemokines such as IL-6 and IL-8 [116]. Recent evidence has also demonstrated that the cyclic GMP-AMP synthase (cGAS)-stimulator of interferon genes (STING) signaling pathway, a critical marker of the innate immune response, stimulates and regulates the SASP as a consequence of the accumulation of cytoplasmic DNA (cytoplasmic chromatin fragments, mtDNA, and cDNA) in senescent cells [117].

The SASP exerts both autocrine and paracrine functions that influence senescent cells, but also neighboring cells, particularly innate and adaptive immune cells, which eventually result in the clearance of senescent cells and contribute to the final tissue hemostasis [118]. In an autocrine fashion, the SASP often reinforces senescence through factors such as CXCR-2 [119] and IL-6 [120] that can bind to self-receptors. In contrast, the SASP’s paracrine effects promote tumor suppression by the activation of either the p53/p21^Cip1^ or p16^INK4a^/Rb tumor suppressor pathways; at the same time, the SASP can drive the progression of premalignant cells to develop more aggressive phenotypes through, for example, the stimulation of epithelial-to-mesenchymal transition (EMT) [30]. Evidence also indicates that the SASP of senescent fibroblasts enhances proliferation and facilitates the malignant transformation of pre-malignant epithelial cells along with tumor vascularization; events mediated by the proinflammatory cytokines IL-6 [121], IL-8 [122], vascular endothelial growth factor (VEGF) [123], and the matrix metalloproteases (MMPs) [124], while inducing senescence in adjacent non-senescent cells as a bystander effect in a manner that contributes to the heterogeneity of premalignant lesions. Collectively, the SASP in general opposes the cell-autonomous tumor-suppressing function of senescence and allows senescent cells to contribute to chronic inflammation, aging processes, and cancer.

### 2.2. Evidence for Oncogene-Induced Senescence (OIS)

Genomic instability confers high tumorigenic potential and increases the risk of malignant transformation. Since eukaryotic cells undergo senescence as a consequence of telomere shortening/dysfunction, as mentioned previously, in essence, senescence reflects a tumor suppressive mechanism, in that it facilitates the suppression of further proliferation of cells with the potential of becoming cancerous [8]. In a similar fashion, OIS serves as a classical example of a form of senescence that is primarily induced in response to tumorigenic events. Specifically, OIS is a robustly brought about by aberrant activation of oncogenic signaling, which is generally driven by activating mutations of oncogenes such as Ras, Akt, E2F1, B-Raf, and cyclins [92,125], or the inactivation of tumor suppressor genes such as PTEN and NF1 [11]. One of the first observations of OIS was reported by Serrano et al., who showed that the insertion of an activated Ras allele (H-Ras V12) into primary diploid fibroblasts resulted in the induction of cellular senescence hallmarks including p53 and p16^INK4a^ accumulation, an enlarged, flattened morphology, as well as suppressed mitotic activity [60]. These in vitro findings were later supported by Sarkisian et al. using in vivo models, further demonstrating that oncogene expression levels appeared to be critical for OIS induction [126]. In these studies, Sarkisian et al. utilized doxycycline-inducible transgenic mice that permit the “titration” of Ras activation, demonstrating that cellular proliferation as well as mammary epithelial hyperplasia, were stimulated by low levels of Ras activation; however, senescence required the establishment of high levels of Ras activation [126].

Several oncogenes have been associated with the induction of senescence (Table 2). In addition to Ras, a GTPase, first established to induce OIS by Serrano et al. [60], and later became the most frequently utilized oncogene to induce senescence experimentally, others have demonstrated that B-Raf, a serine/threonine-protein kinase, can induce a p16^INK4a^-driven senescent growth arrest in cells found in Spitz nevi (benign melanocytic precursors for melanoma) [10,127]. Hyperactivation of PI3K/Akt pathways have also been demonstrated to result in senescence induction through the promotion of p53 [128]. Overexpression of cyclins, such as cyclin E, has also been shown to promote OIS marked by SA-β-gal expression and p16^INK4a^ upregulation [92]; that is, cells activate a senescence program to delay or interfere with the tumorigenic actions of drivers of the cell cycle. As is the case with oncogene overexpression, the inactivation of some tumor suppressor genes can also force somatic cells into a senescent growth arrest. For example, loss of the PTEN can precipitate p53-dependent OIS that prevents further cellular proliferation, and interferes with initial malignant transformation in vivo [129].

Oncogene-induced senescent cells have been shown to accumulate in premalignant lesions, which by definition, are rich in cells harboring mutations that either result in oncogene hyperactivation or tumor suppressor gene inactivation. For example, senescent cells accumulate in dysplastic skin and oral mucosal lesions [130]. Evidence for OIS has also been established in several other premalignant processes including human preneoplastic gastrointestinal lesions [92,131,132], pre-melanoma nevi [133], prostatic intraepithelial neoplasia [77], oral leukoplakia [134], and premalignant nasopharyngeal epithelium [135]. In premalignant lesions, OIS is thought to act as a “failsafe” protective mechanism, limiting the propagation of cells harboring oncogenic DNA [129]. For instance, high levels of Ras activation in mammary epithelial cells resulted in senescence induction in vivo and promoted the induction of tumor-suppressive pathways [126]. Inactivation of PTEN resulted in the promotion of p53-dependent senescence and prevented the onset of aggressive prostatic cancer [129]. Furthermore, induction of senescence increases the susceptibility to immune clearance, since both components of the adaptive and innate immune cells have the capacity to recognize and remove the deleterious accumulation of senescent cells [136,137,138]. Activation of DDR responses and senescence induction can act as barriers to malignant transformation, and cancer progression may rely on bypassing these checkpoints. Evidently, mutational inactivation of key DDR genes can drive the development of human somatic cancers [139] and frequently alters cellular susceptibility to senescence and apoptosis [140], allowing evasion of regulatory and cell death pathways.

**Table 2:** Examples of established oncogene/tumor suppressor genes implicated in the induction of Oncogene-Induced Senescence (OIS).

Oncogene/Tumor Suppressor Gene	Alteration	Function	Model	Premalignant/Malignant Lesion	Reference
c-mos	Overexpression	Serine/threonine kinase	Human fibroblasts	Lung cancer	[92]
PTEN	Loss of function	Tumor suppressor gene	Murine embryonic fibroblast	Prostate cancer	[129]
Ras	Activation	Regulation of signal transduction	Murine embryonic fibroblast	Pancreas, colon, and lung cancers	[60]
Raf	Activation	Ras signaling	Human diploid fibroblast	Lung adenomas	[141]
Akt	Activation	Akt signaling	Murine embryonic fibroblasts Endothelial cells	-	[142]
E2F1	Overexpression	Promotes G1 to S-phase	Human diploid fibroblasts	Pituitary gland hyperplasia	[143]
Cyclin E	Overexpression	Activation of cyclin dependent kinase-2	-	Breast cancer	[92]
E7	Overexpression	Inactivation of Rb	HCA2 human fibroblasts	-	[18]

In contrast to the anti-tumorigenic role of OIS, components of the SASP can promote tumorigenicity. SASP factors are highly variable and can enable both a tumor-suppressive and a tumor-promoting environment [144,145,146]. Depending on the spectrum of secreted factors, SASP factors can promote tumorigenesis through the activation of proliferation pathways, immunosuppression, and promotion of a migratory phenotype [147,148,149]. For example, secretion of specific SASP factors, such as IL-6 and IL-8, exert paracrine effects on surrounding cells and promote tumorigenesis in vivo [147,150]. Moreover, studies in a pancreas model showed an OIS-mediated tumor-promoting effect where the elimination of OIS cells decelerated the development of pancreatic cancer in animals [12]. These studies and others delineate the heterogeneous nature of OIS in different models, which may be mediated through different combinations of downstream effectors as well as the interplay between senescent cells and the microenvironment [10,92,129]. Thus, whether senescence induction is beneficial or harmful with regard to tumorigenesis is not fully understood and may be contextual in nature. Importantly, since HPV infection can induce OIS in somatic human cells, it is likely that the accumulation of HPV-positive senescent cells in cervical or head and neck premalignant lesions could contribute directly to tumorigenesis.

## 3. Human Papilloma Virus (HPV)-Induced Senescence

Human papillomavirus (HPV) infection is the most common sexually-transmitted infection among women globally [151]. Multiple clinical and epidemiological reports have clearly established the role of HPV infection as the primary etiologic factor for cervical cancer [152]. Furthermore, HPV infection is associated with cancerous transformation in the penis, vagina, anus, vulva, and oropharynx [153]. Of the fifteen high-risk (HR) HPV genotypes that can cause cancer at these sites, HPV 16 and 18 are the most common, causing 70–75% of cancer-associated lesions in humans [154]. Low-risk (LR) HPV genotypes 6 and 11 are rarely associated with cancer, and are instead more closely associated with warts and respiratory papillomatoses [155]. The oncogenic HPV is a double-stranded DNA virus that infects epithelial cells in the anogenital region or naturally discontinuous oropharyngeal epithelium. Most HPV infections remain asymptomatic and are cleared by the immune system in the 6–18 months after infection. Only a minority of infected patients fail to eliminate the infectious virus and, after a latency period, develop dysplastic changes such CIN.

HPV replication in target cells leads to the production of the early (E1–E7) and late proteins (L1, L2). The major and minor capsid proteins L1 and L2, respectively, enclose the HPV genome [156,157]. The virus capsid is made up of L1 protein pentamers, while virus-like particles, which are extremely protective and produce large amounts of neutralizing antibodies, are created when the L1 and L2 proteins are self-assembled [158,159]. The HPV E1 is the only viral protein exhibiting enzymatic activity in the Human Papillomavirus family, and its major known function is to control the viral DNA replication process [160]. HPV E2 is essential for viral genome replication, RNA transcription, and viral epigenome partitioning during replication [161,162]. HPV E4 has been demonstrated to cause G_2_/M arrest and aid in the amplification of E6/E7 viruses; it also contributes to viral genome amplification, and the maintenance of MAPK activity, and may interact with and stabilize E2 [163,164,165]. It has been demonstrated that HPV16 E5 cooperates with E7 in cell transformation, inhibits immunological response, and increases cell motility [166]. Importantly, the oncogenicity of HPV is primarily attributed to the oncogenic proteins E6 and E7, which interfere with select cell signaling pathways and continue to be expressed during tumor formation and progression [16]. E6 and E7 enhance cellular transformation through the inactivation of the tumor-suppressor proteins, p53, and Rb protein, respectively, which results in cell cycle disruption and the accumulation of DNA mutations [19].

While the tumor-suppressive aspect of senescence has been established in response to DNA aberration-associated telomere dysfunction or oncogene hyperactivation, its contribution to the suppression of virus-mediated transformation is not fully understood. A seminal finding by Baz-Martínez et al. indicates that senescence interferes with the replication, and thus the infectivity, of vesicular stomatitis virus (VSV) in several cell models including MEFs, MCF-7 breast and A549 lung tumor cells [167], strongly suggesting that senescence has antiviral properties. However, in these experiments, senescence was induced by classical means, e.g., replicative exhaustion or DNA-damaging agents, followed by viral infection. Thus, there was no examination of whether senescence is directly induced in somatic fibroblasts by VSV infection. Conversely, another report by Kim et al., presents opposing evidence. As in the work of Baz-Martinez, senescence was induced in primary human bronchial epithelial cells (HBE) and human dermal fibroblasts (HDF) through replicative exhaustion, which was followed by viral infection using influenza virus (IFV) and varicella-zoster virus (VZV) [168]. Unexpectedly, viral replication was *enhanced* in senescent cells in comparison to their non-senescent counterparts. That is, senescent HDF exhibited approximately 1.5-fold higher VZV infectivity titers than non-senescent HDF. Moreover, the expression levels of VZV glycoprotein E were higher in senescent cells, indicative of higher replication [168]. These findings highlight the complexity of considering senescence as an antiviral defense mechanism.

The first report to hint that HPV infection might be associated with senescence induction was provided in studies by Velasco et al., which described p53 upregulation in HPV-positive cells [169]. Subsequently, the Shay laboratory showed that HPV16 E6 and E7, through their modulation of the tumor suppressors p53 and pRb, are necessary for human diploid fibroblast cells to evade cellular senescence [170,171]. Interestingly, replicative senescence was reversed in cultures of human skin fibroblasts by the ability of E6 oncoprotein to inactivate p53 [172]. Accordingly, the hypothesis at the time was that E6 and E7 are key factors for premalignant replicatively exhausted cells to escape from senescence [173]. In later studies, the Howley laboratory demonstrated that the artificial expression of E2 in HPV-positive cancer cells results in a senescent growth arrest, accompanied by robust p21^Cip1^ upregulation, which can be reversed through the re-expression of exogenous E6 and E7 [174,175]. E2 blocks the transcription of E6 and E7 [176]. Importantly, these studies were performed in HeLa cells which are HPV positive and possibly addicted to E6 and E7 for their immortalization; thus, repression of E6 and E7 resulted in a senescent growth arrest [177,178,179]. As an alternative to using E2, these results were confirmed using shRNA-mediated knockdown of E6 and E7 expression, which also caused senescence-mediated growth arrest in HeLa cells [180]. Moreover, pharmacological inhibition of E6-mediated degradation of p53 results in a reduced proliferative capacity of HPV-positive cancer cells and senescence induction [181,182]. Lastly, CRISPR/Cas9-mediated knockout of E6 or E7 induced senescence in HPV 18-immortalized HeLa cells, marked by cellular hypertrophy, upregulation of SA-β-gal, and Lamin B1 degradation [183].

In marked contrast to the studies described above, transfection of naïve somatic cells (i.e., not previously infected with HPV) with the HPV oncoprotein E7 has been shown to induce senescence (e.g., transfecting WI-38 human fibroblasts with E7) [184]. Moreover, Rodier et al. have confirmed the use of E7 as a senescence inducer in HCA2 human fibroblasts, similar to Ras-induced senescence models, and showed that ectopic E7 expression results in significant SA-β-gal expression and formation of DNA-SCARS [18]. Moreover, cancer-associated fibroblasts (CAF) were found to secrete high levels of IL-6 upon STAT3 activation and exhibit a senescence morphology in vitro or in cervical cancer tissues infected with high-risk HPV, where the overexpression of E6 activates STAT3, increases IL-6 expression and induces senescence [185]. Importantly, it is well-known that clinically-isolated samples of the cervical epithelium (using Pap smears) have high levels of p16^INK4a^ (and p14^ARF^) expression, suggesting that early during HPV infection, cervical epithelial cells are in a senescent state [186]. More specifically, Feng et al. investigated the protein expression levels of p14^ARF^, p15^INK4b^, p16^INK4a^, p53, and Ki-67 in a tissue microarray of 20 samples of CIN (II-III) [187] and reported a significant upregulation of the senescence-associated markers p14^ARF^, p15^INK4b^, p16^INK4a^ in CIN compared to their normal cervical epithelial counterparts, suggesting that cervical premalignant lesions are highly enriched in senescent cells [187]. However, given that Feng et al. did not assess the HPV infection status in the CIN samples, it may prove to be the case that the majority were HPV positive, since HPV is the main oncogenic driver of cervical premalignant lesions. If this were, in fact, the case, accumulation of senescent cells in CIN lesions should provide an avenue whereby pharmacological therapy can be utilized to selectively target cervical senescent cells and prevent or delay malignant progression.

**Figure 1:**
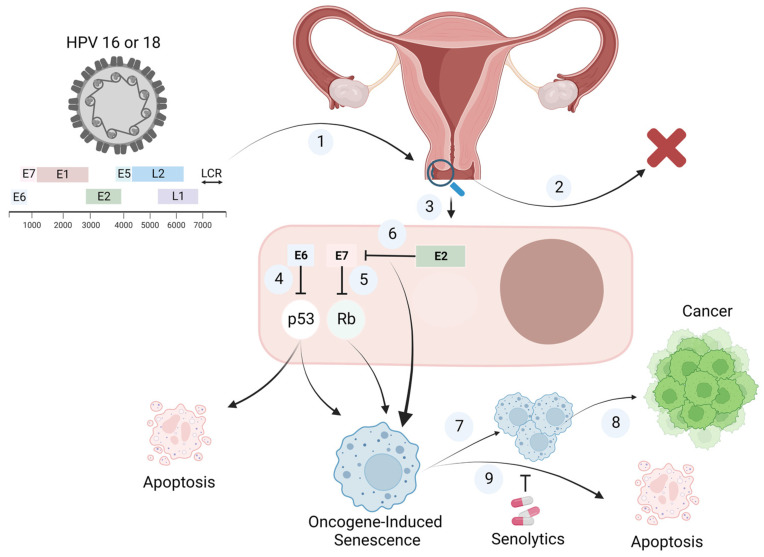
Proposed Model for The Contribution of HPV-Induced Senescence to Cervical Cancer. Oncogenic variants of the Human Papilloma Virus (HPV) infect basal squamous epithelial cells of the cervix ①. Fortunately, the majority of infections resolve spontaneously and are cleared within two years ②. however, approximately 1% of infected patients can develop cervical cancer ③. The oncogenic potential of HPV is primarily attributed to the oncoproteins E6 and E7, which lead to the degradation/inactivation of the tumor suppressor genes p53 ④ and ⑤ Rb, respectively. As a defense mechanism, several HPV-infected cells undergo apoptosis and are eliminated. Alternatively, cervical epithelial cells may undergo senescence as a tumor suppressor mechanism. Primarily, cells that have become dependent on E6- or E7-mediated suppression of cell cycle control can undergo senescence as a consequence to E2-mediated inactivation of E6 or E7 ⑥. Otherwise, when a naïve, virus-free cell is infected with HPV, cells undergo senescence, which is likely to represent an Oncogene-induced Senescence (OIS) variant. Oncogene-induced senescent cells infected with HPV accumulate as a component of the cervical premalignant lesion (CIN) being generated ⑦. Only those cells that manage to escape OIS or those under constant pro-tumorigenic stimulation by the SASP, are capable of progressing into malignant phenotypes ⑧. Alternatively, we propose the use of senolytics, compounds that selectively eliminate senescent cells, to interfere with the accumulation of premalignant senescent cells in cervical lesions, as a novel pharmacological approach to interfere with the development of HPV-induced cervical cancer ⑨.

Collectively, our literature review suggests that, while most studies indicate that inactivation of the HPV oncogenic proteins E6 or E7 is the primary inducer of senescence, these studies were conducted predominantly in immortalized cells that had previously been infected by the HPV virus and were likely dependent on E6 or E7-mediated inhibition of p53 and Rb. On the other hand, it appears that when a naïve, virus-free cell is infected with HPV (of course, restricting this hypothesis to oncogenic variants 16 and 18), cells undergo senescence, which is likely to represent one form of OIS. This suggests that cells that have already been immortalized by HPV infection through the expression of E6 or E7 can still undergo senescence upon the suppression of these oncoproteins, and in addition, cells that have not been exposed to HPV can undergo senescence once infected due to the ability of E7 to induce OIS. Consequently, our hypothetical model suggests that cervical epithelial cells infected with HPV undergo one form of senescence in the process of developing clinically detectable premalignant lesions (CIN), or at least, senescence represents a component of those premalignant lesions. However, only those cells that manage to escape OIS or those under constant pro-tumorigenic stimulation by the SASP, are capable of progressing into malignant phenotypes (Figure 1).

## 4. Should Senolytics Be Considered for the Elimination of HPV-Infected Senescent Cells?

Senolytics are a diverse group of natural and synthetic compounds that have been found to selectively kill senescent cells. Initially, senolytics were identified through high-throughput drug screening designed to identify compounds that can eliminate senescent cells induced by replicative exhaustion [188]. Therefore, the translational goal for the development of these compounds was to cull senescent cells that accumulate during organismal aging to mitigate some of the senescence-associated aging-related pathologies [189]. Preclinical evidence has demonstrated the efficacy of senolytics in eliminating senescent cells in vitro, but more importantly, in eliminating senescent cells in animal models of atherosclerosis [190], osteoarthritis [191,192], neurodegeneration [193,194], neuropathy [195], fibrotic lung disease [196], and chronic kidney disease [197], among many others. Moreover, early evidence from clinical trials investigating some of these compounds in aging-related disorders has generated cautious optimism [198]. Of several senolytics being investigated clinically, the most successful ones that are currently being studied in the clinic include the dasatinib + quercetin cocktail [199,200], fisetin [NCT04815902, NCT04210986, NCT03325322], and inhibitors of members of the BCL-2 family, particularly, BCL-X_L_ [NCT04229225, NCT04129944, NCT04537884, NCT04857996].

Senolytics have also been proposed as adjuvant cancer treatments [201], since therapy-induced senescence represents a major component of tumor biology and an established outcome of cancer therapeutics [68,202]; in this context, senolytics have shown significant efficacy in eliminating senescent tumor cells both in vivo and in vitro [71,106,203,204,205]. In addition to reducing tumor volume, senolytics have also been shown to reduce the ability of tumor cells to metastasize [106], interfere with therapy resistance [71,204,206], and alleviate some of the therapy-associated adverse effects of chemotherapy [207]. Thus, senolytics have a substantial utility in cancer therapy, despite several limitations including their heterogenous effect among different cancer models and some having significant toxicity when employed in vivo [208]. As such, efforts continue to identify more efficacious and safe compounds that exert senolytic potential and can be exploited for cancer treatment [204].

Despite having the promising potential to eliminate senescent cells induced by replicative exhaustion (RS) or exposure to various therapeutics (TIS), there is currently less evidence available relating to the ability of senolytics to eliminate oncogene-induced senescent cells (OIS) as a strategy for treating premalignant lesions where oncogene-induced senescent cells represent a major component [21]. As we propose in this work, HPV can also induce a form of OIS and has a role in the development of cervical premalignant lesions, and senolytics can also be investigated for the treatment of CIN and other premalignant lesions where HPV plays a pathogenetic role.

Several studies support the potential feasibility of senolytics to eliminate OIS. For example, the overexpression of H-Ras in WI-38 human fibroblasts renders these cells susceptible to killing in vitro by the BCL-2 inhibitor, navitoclax (ABT-263) [209]. In a similar fashion, navitoclax (ABT-263) was shown to eliminate senescent cells in a KIAA1549:BRAF fusion-driven, pilocytic astrocytoma DKFZ-BT66 cell model [210,211]. In addition to navitoclax, natural compounds such as piperlongumine [212] have been shown to kill oncogene-induced senescent cells; however, the frequently used dasatinib + quercetin cocktail has not proven to be similarly effective [211]. Ouabain, a cardiac glycoside, that has been shown to eliminate senescent cells induced by replicative exhaustion or therapy, was also reported to kill senescent cells induced by a transposon-mediated transfer of oncogenic N-Ras in vivo [213]. Moreover, ouabain was shown to exert comparable senolytic activity in a mouse model of adamantinomatous craniopharyngioma [214]. However, a profound limitation of ouabain as a senolytic stems from the fact that the investigated concentrations used to demonstrate its senolytic ability are supraclinical, but with the exception of a report by L’Hôte et al. where ouabain in the nanomolar concentration ranges eradicated oncogene-induced senescent cells [215].

To more directly interrogate the overall effect of the senolytic-mediated elimination of oncogene-induced senescent cells, Kolodkin-Gal et al. utilized a model of K-RAS- induced pancreatic adenocarcinoma, where K-Ras overexpression led to the formation of premalignant pancreatic lesions [12]. Treatment of mice harboring these pancreatic lesions with ABT-737, another BCL-2 inhibitor, and an established senolytic, resulted in a dramatic reduction in the burden of senescent cells in the pancreas and was accompanied by a decreased expression of several SASP factors [12]. Senolytic elimination of oncogene-induced senescent cells reduced the chance of the progression of pancreatic premalignant lesions into fully transformed pancreatic adenocarcinoma. [12]. Whether the elimination of HPV-induced premalignant cells in cervical or head and neck tissue would result in a similar outcome remains largely unknown.

There is no direct evidence for senolytics having been tested in HPV-induced senescence models; however, recent studies have indicated the potential utility of senolytics for the treatment of illnesses caused by other viral infections, including SARS.CoV.2 [216,217,218]. Preliminary data by Pham et al. indicated that Merkel cell polyomavirus is capable of inducing senescence in human skin fibroblasts coupled with a robust SASP; here the senolytic, navitoclax, decreased senescence and viral genome levels in these cells [219]. This led to the proposition of utilizing senolytics as antiviral therapy [220]. Moreover, a review of the recent literature has led Giannakoulis et al. to hypothesize that the use of senolytics might be beneficial in interfering with HBV and HCV oncogenic potential, since viral senescence appears to contribute to the development of hepatocellular carcinoma [221]. Furthermore, Szaniawski et al. have proposed the use of senolytics in HIV-1 persistence and HIV-1-associated immune exhaustion driven by the accumulation of senescent cells [222]. Lastly, the most direct evidence in support of this review is on the use of metformin, a senomorphic rather than a senolytic, wherein metformin blocked senescence induction in HPV-positive cancer cells in response to E6/E7 inhibition, allowing HPV-positive cancer cells to escape from therapy-induced senescence [223]. While this escape from senescence might not be a desirable outcome, it provides proof of concept that HPV-induced senescence is amenable to modulation by several of the currently available senolytics and senomorphics. Collectively, the evidence thus far available in the literature supports the need for further studies to test whether exploiting HPV-induced senescence as a target for established or novel senolytics might be valuable for developing pharmacological strategies for the prevention of virus-induced premalignant/malignant transformation.

## Figures and Tables

**Table 1:** Examples of the most frequently described hallmarks of cellular senescence.

Hallmark	Description	Reference
Growth arrest	Upregulation of p21^Cip1^	[32]
	Upregulation of p16^INK4a^	[33]
	Downregulation of ribosomal biogenesis	[34,35]
Morphological changes	Large and flattened appearance	[36]
Suborganellar damage	Telomere dysfunction	[37]
	Persistent activation of the DNA damage repair response (DDR)	[28]
	DNA-SCARSs	[18]
	Proteosomal activity	[22]
	Accumulation of reactive oxygen species (ROS)	[38]
	Enhanced lysosomal biogenesis (SA-β-galactosidase)	[26]
	Accumulation of protein aggregates (lipofuscin)	[39,40]
	Mitochondrial dysfunction	[41]
Epigenetic changes; SAHF	Histone edits (H3K9Me3, HP-1, γH2AX)	[42,43]
The SASP	Growth factors	[44]
	Chemokines	[29]
	Cytokines	[45]
	Angiogenic factors	[46]
